# Extracellular vesicles: how they interact with endothelium, potentially contributing to metastatic cancer cell implants

**DOI:** 10.1186/s40169-017-0165-2

**Published:** 2017-09-21

**Authors:** Murray M. Bern

**Affiliations:** University of New Mexico Comprehensive Cancer Center, 1201 Camino de Salud, Albuquerque, NM 87131 USA

**Keywords:** Extracellular vesicles, Microparticles, Exosomes, Endothelium, Metastatic carcinoma, Hypercoagulation, Tissue factor, P-selectin, P-selectin glycoprotein ligand 1

## Abstract

Extracellular vesicles (EV) are blebs of cellular membranes, which entrap small portions of subjacent cytosol. They are released from a variety of cells, circulate in the blood for an unknown length of time and come to rest on endothelial surfaces. They contribute to an array of physiologic pathways, the complexity of which is still being investigated. They contribute to metastatic malignant cell implants and tumor-related angiogenesis, possibly abetted by the tissue factor that they carry. It is thought that the adherence of the EV to endothelium is dependent upon a combination of their P-selectin glycoprotein ligand-1 and exposed phosphatidylserine, the latter of which is normally hidden on the inner bilayer of the intact cellular membrane. This manuscript reviews what is known about EV origins, their clearance from the circulation and how they contribute to malignant cell implants upon endothelium surfaces and subsequent tumor growth.

## Definition of extracellular vesicles

Cells shed blebs of their phospholipid bilayer plasma membranes as byproducts of cell growth, apoptosis and in response to physiologic and pathophysiologic stimuli. These vesicles encapsulate small portions of the subjacent cytosol, creating a heterogeneous population of phospholipid-walled vesicles. These particles are referred to as extracellular vesicles (EV), but also as microparticles, microvesicles, microsomes, lipid vesicles, apoptotic blebs and exosomes [1–6].

EV’s are characterized by their size (30–100 nm for exosomes and 100–1000 nm diameter for the larger microvesicles), by their cells of origin including megakaryocytes, platelets, red blood cells, endothelial cells and others, and by their intravesicular contents [2, 3, 6, 7]. Their intravesicular contents depend upon their cells of origin and can include tissue factor, double stranded DNA, mRNA, microRNA, adhesion integrins, growth factors, protease inhibitors, P-selectin glycoprotein ligand-1 (PSGL-1) and ceramides [8–17]. They have been detected in blood plasma and other physiologic fluids [3, 4]. Gender, age of subjects and diseases influence their number in circulating blood and their size distribution [18, 19].

EVs are also released in response to pathophysiologic stimuli including thrombin, shear stress, complement activation, sepsis, hypoxia, inflammation, from malignant cells and following chemotherapy for malignancies [20–22]. They may also be byproducts of cell maturation with shedding of excess cell membrane [23].

**Fig. 1:**
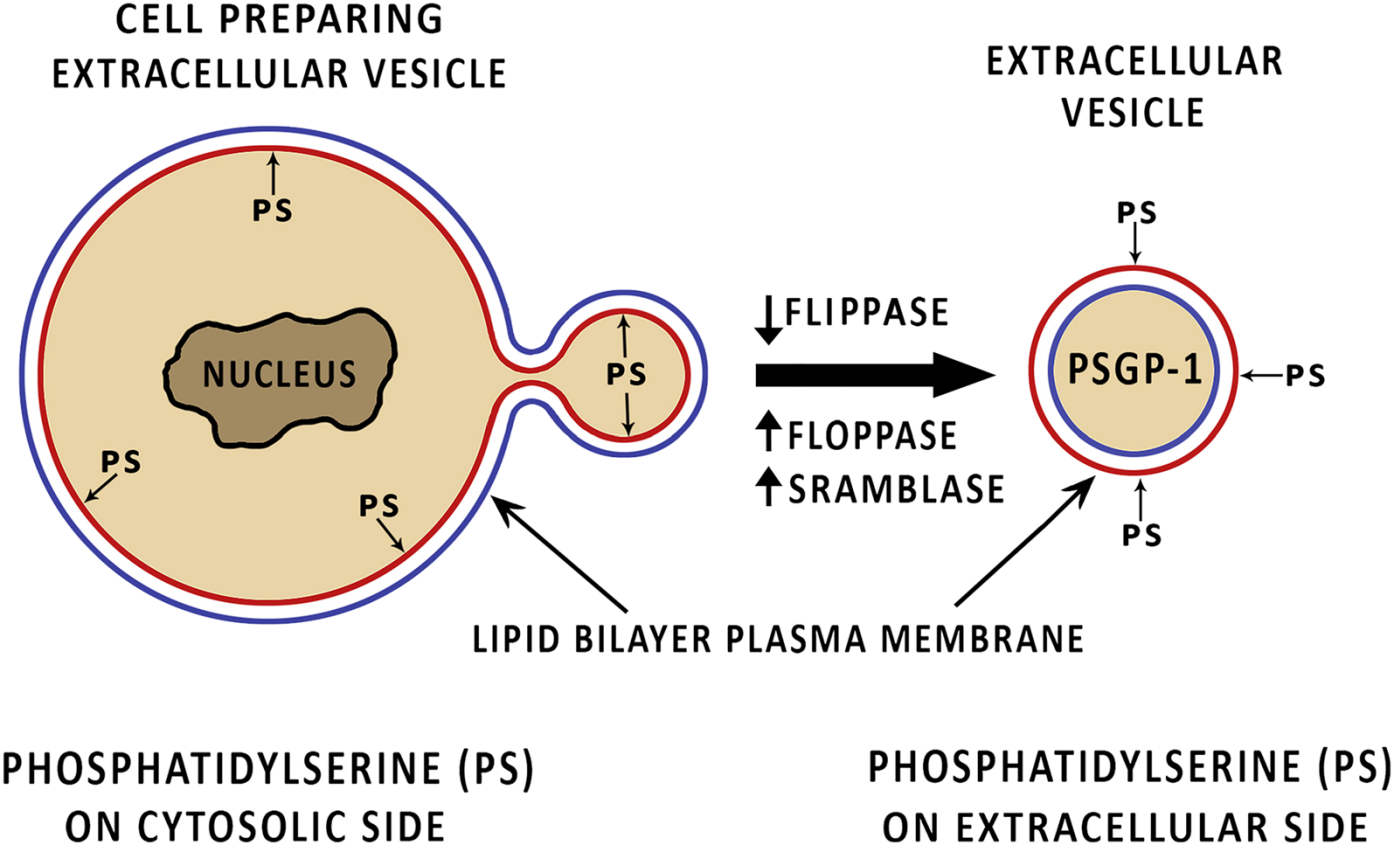
The normal cell membrane is an asymmetrical bilayered structure with phosphatidylserine- and phosphatidyl-ethanolamine-enriched cytosolic layers, maintained by flippase, floppase and scramblase. As extracellular vesicles are formed, the flippases is inactivated while floppase and scramblase are activated, leading to reversal of the normal asymmetry, creating an outward facing phosphatidylserine enriched layer

**Fig. 2:**
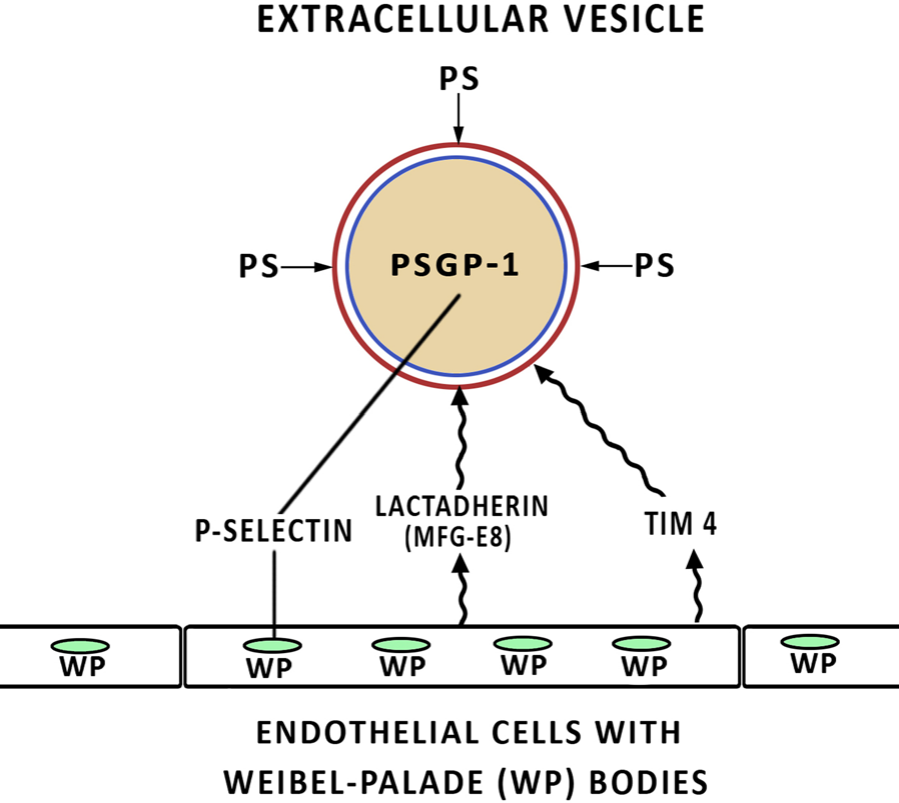
Extracellular vesicle attachment to endothelial cells is dependent upon their exposed PSGL-1 attaching to the P-selectin expressed from Weibel–Palade bodies and platelet alpha-granules, and upon the tethering of the exposed surface phosphatidylserine to Tim4, lactadherin/MFG-E8 and probably other cell adhesion molecules

The normal cell membrane is a bilayer structure with inner cytosolic layer enriched with phosphatidylserine and phosphatidyl-ethanolamine. This structure is maintained by enzymes flippase, floppase and scramblase [6]. When the EV are created, the endoplasmic reticulum releases Ca^2+^ which inactivates flippase and activates floppase and scramblase leading to loss of the normal asymmetry of the cell membrane and reversal of the normal order, creating an outward facing phosphatidylserine enriched layer [6, 24]. See Fig. 1. The phosphatidylserine is then available to be tethered by lactadherin (also known as Human Milk Fat globule factor 8 or MFG-E8) and Tim 4 to endothelium [25, 26]. See Fig. 2.

Exosomes are unique subsets of EVs with specific biogenesis, measuring 30–100 nm [4, 17]. They are derived from endosomal multivesicular bodies, which then fuse with plasma membranes and are secreted from the cell into extracellular space, a process termed exocytosis. The primary function of these endosomes appears to be for the cellular uptake and extrusion of macromolecules from and to the extracellular fluids [1–3, 27]. They transport bioactive molecules including proteins, DNA, functional mRNA and microRNA from cell to cell by membranous transfer, thereby influencing other cell systems [17, 25, 28]. The currently available techniques do not allow for clean separation of these exosomes from the larger extracellular vesicles.

Apoptotic bodies may co-isolate with extracellular vesicles when isolated in the lab, but based upon origin they differ greatly from EV [29]. The EV appear to be generated continuously from normal, viable cells, while the apoptotic bodies are derived following a highly regulated programmed cell death, in which the caspase pathway plays a central role. Apoptosis involves cell shrinkage and nuclear fragmentation, with the debris separated into nuclear or cytoplasmic vesicles of various sizes, which may be as small as the EV, but also may be up to 5000 nm diameter. They do express the “eat me” cellular signal to facilitate their phagocytosis by macrophages in the vicinity of the dead cell, using some of the same signals as the other EV [29].

## Methods of isolation

The methods of isolation and preservation of the EVs are improving, but have not reached the ultimate goal of allowing analysis of homogeneous EV subpopulations and precise study of their targets [3–5, 30]. Currently EVs are defined predominantly by the techniques used for their isolation (flow cytometry, density gradient separation, differential centrifugation, size-exclusion chromatography, immunoaffinity isolation, polymer precipitation and others) as they affect the size and density of the isolates. Also, pre-analytical manipulation of samples influence the study results [31]. As of now there is overlap of the size subclasses and cells of origin [4, 5]. For reporting purposes the EV can be further described by flow cytometry, mass spectroscopy and by their specific contents such as DNA, microRNA and mRNA [3, 32, 33].

A new technique for isolation of EV was reported using affinity-based methodology taking advantage of the T cell immunoglobulin domain and mucin-containing protein 4, known as Tim4, and its adherence to the externalized plasma membrane phosphatidylserine [34]. In this method the extracellular domain of Tim4, normally expressed as a transmembrane protein on macrophages, is immobilized on magnetic beads. Since Tim4 strongly binds to phosphatidylserine it immobilizes the EV on the beads [35]. This binding is calcium dependent and thus EV can be released when calcium chelators are added. Tim4 affinity can also be used in ELISA and flow cytometry formats [34].

Post-translational modifications of EV proteins are under study [3, 36, 37]. Exosomes become more rigid increasing their stability after they are released from m into the extracellular space with its increased pH. They also undergo post-translational covalent attachment to fatty acids. Acetylation and myristoylation promote protein sorting and subsequent EV budding [36, 38].

## Total mass of EVs in circulation

The total mass of EVs in the circulation at any given time can be represented by their rates of release into the circulation and their rates of clearance from the circulation. How long they survive in the circulation is unclear [33]. Clearance mechanisms include endocytosis, micropinocytosis, phagocytosis and membrane fusion [22, 39]. These processes are dependent upon exposed PSGL-1 on platelet-derived EV attaching to P-selectin expressed from the endothelial cell Weibel–Palade bodies and platelets alpha-granules; and upon the phosphatidylserine on their surface with tethering to Tim4 and lactadherin/MFG-E8 [25, 40]. The extent of surface phosphatidyl serine exposure in-vivo prior to ex vivo manipulation is unknown.

Their rates of clearance have acute and chronic patterns of change and appear to be influenced by their cell of origin [22, 41, 42]. The locales of the macrophage-mediated phagocytosis also differ based upon the organ in which the macrophages are anchored [43–45]. Endothelial receptors may also vary from site to site with different organ-specificities, such as the endothelial cell-rich lungs [46, 47].

## EV activities

The total array of activities of the EVs is still under study. They are involved in cell-to-cell communications, transferring their contents to cell types that differ from their cell of origin [9–12]. This affects cell signaling, platelet and leukocyte adhesion to subendothelial matrix, vascular smooth muscle proliferation, inflammation, immune response, thrombosis, angiogenesis and angiocrine activities, and tumor niche formation leading to enhanced tumor implantation on endothelial surfaces [4, 47–55].

### Contributions to thrombosis

An important and complex picture is emerging of how EVs participate in coagulation and fibrinolysis [8, 12, 49, 56–58].

EVs become anchored to endothelium in two ways. The PSGL-1 from platelet-derived EV attaches to the P-selectin released from Weibel–Palade bodies of the activated endothelial cell and from the platelet-derived α**-**granules [55, 59–61]. There also appears to be a phosphatidylserine receptor, at least on cultured activated microvascular endothelial cells derived from pulmonary and retinal tissues [62]. CD36, also known as platelet glycoprotein IIIB and IV among other names, is a scavenger receptor that binds several ligands including phosphatidylserine. It is a candidate cellular adhesion molecule for anchoring the EV. This pathway is blocked when the phosphatidylserine as pretreated with annexin V [63]. Subendothelial thrombospondin is another candidate cellular CAM for the exposed phosphatidylserine [64]. Other cellular adhesion molecules with ability to tether the phosphatidylserine exposed on the surface of the EV include Tim4 and lactadherin [25, 26].

EV trigger thrombosis via release of tissue factor they contain, supplementing the tissue factor released from perivascular tissue following endothelial disruption [9, 11, 12, 50, 65]. The released tissue factor is activated by thiol isomerases, with downstream platelet activation and thrombus formation [56, 57, 66, 67]. This has become a target of a new class of anticoagulants [68]. As an example, quercetin-3-rutinoside suppresses this tissue factor activation [56, 68]. Furthermore, the exposed phosphatidylserine is a catalytic surface for assembly of the prothrombinase complex [69].

Increased thrombin-generating activity has been recorded for EV derived from platelets and monocytes harvested from patients having recurrent thrombosis [70].

Suppression of tissue factor with anti-tissue factor antibody and the suppression of P-selectin in PSGL-1 knock out mice reduce clot formation [12, 56, 58]. Similarly, drugs with activity in this domain, such as the HMG-CoA reductase inhibitors, suppress the levels of soluble P-selectin and would consequently be expected to reduce the expression of tissue factor [71–74]. Also, the in vitro blockade of the phosphatidylserine on EVs with annexin V or lactadherin (also known as MFG-E8), both of which attach variably to phosphatidylserine, reduces the platelet-derived EVs contribution to thrombin generation [75–78]. These observations are the basis for new clinical studies.

EVs also accelerate fibrin polymerization, supporting the formation of denser clots that are more resistant to fibrinolysis by tissue plasminogen activator (tPA). Platelet-derived EV also attach to the fibrin fibers [79, 80]. When EVs are removed from the test sample the final clot formation is slowed, with reduced fibrin polymerization leading to increased fibrinolysis in response to added tPA [80].

### Extracellular vesicles and malignancies

The complex relationship of EV and cancers is under investigation. These investigations point roles of EV in the metastatic process, cancer progression, drug- and radiotherapy-resistance and in cancer-related hypercoagulation [16, 48, 52–54, 81–86].

EVs derived from ovarian cancer, prostate cancer and fibrosarcoma paradoxically have the potential of potentiating fibrinolysis [8, 87–92]. The EV-associated urokinase plasminogen activator appears capable of promoting invasion of prostate cancer [89]. The EVs that are shed during blood storage loose their fibrinolytic capacity the longer the red cells are stored [92]. It is not yet known how these findings fit into in vivo clotting/fibrinolytic balance or for the metastatic process.

Activated platelets interact with cancer cells by way of P-selectin and the cancer PSGL-1 expressed on malignant cells [93]. Platelet-derived EV promotes tumor growth and tumor-induced angiogenesis [94, 95]. Melanoma cells release exosomes that change the local and systemic microenvironment so as to better support tumor growth and metastasis [52, 54]. They prepare the sentinel lymph node to trap and support growth of the incoming metastatic melanoma cells by inducing lymphangiogenesis in preparation of a premetastatic niche [53, 96]. This process is dependent upon transmembrane proteins. The tetraspanin-integrin complex contributes to the binding of exosomes to their target cells, possibly mediated by P-selectin/PSGL-1 complex [96–99].

As discussed above, tissue factor is activated by protein disulfide isomerases (PDI) [56, 100–104]. It is also known that many cancers are dependent upon protein disulfide isomerase for survival and metastasis [105]. Thus PDI inhibitors may serve both as anticoagulant and as tumor suppressant [68].

The EVs derived from cancer cells appear to carry some specific physiologic activities, including the increased tissue factor [6, 11, 106–115]. This blood-borne tissue factor may contribute to the hypercoagulation syndrome that accompanies many cancers [6, 110, 113–117]. The amount of tissue factor-positive EV correlates with venous thrombosis seen in patients with cancer [7, 113–116]. In another study epithelial cancer cells adopted mesenchymal features upon exposure to activated epithelial growth fact receptor coupled with blockade of E-cadherin, leading to a surge of released EV-containing epidermal growth factor receptor (EGFR) and tissue factor [117]. Upon transfer of the tissue factor to cultured endothelial cells, they become procoagulant [11, 117]. Cancer cell-derived EV carrying PSGL-1 can accelerate thrombus formation in vivo by aggregating platelets via the tissue factor-dependent pathway. Their intact parent cells do not have the same capacity [113, 114]. When microparticles derived from tumor cell are injected into mice acute thrombocytopenia and signs of shock follow. This was prevented by prior heparinization [42]. When exposed to hypoxic conditions tumor cell-derived EVs demonstrate the potential for increasing angiogenesis and facilitating metastasis, leading to changes in cell–cell and cell-extracellular matrix interaction allowing for increased invasiveness [118].

## Further exploration of extracellular vesicle activity on vascular endothelium

Endothelial cells are involved with tumor growth, tumor-induced angiogenesis and angiocrine functions for self-renewal and differentiation following trauma and thrombosis [119, 120].

As demonstrated with phage display, endothelium expresses different receptors depending upon their organ of origin and their functional status [121–125]. This phenomenon has been nicknamed endothelial ZIP codes [126]. This may have therapeutic implications.

Endothelial dysfunction occurs with many pathological states, including sepsis, thrombotic thrombocytopenia purpura, pulmonary hypertension, sickle cell diseases, activation of the complement C5-9 membrane attack complex and exposure to cytotoxic chemotherapy among others [108–110]. These result in changes of phenotype for thrombo-resistance including decreased production of thrombomodulin, tissue-derived plasminogen activator (tPA), heparan sulfate, plasminogen activator inhibitor-1 (PAI-1), but also increased expression of selectins, including over expression of P-selectin from the Weibel–Palade bodies thus leading increased production of procoagulant activity [126]. The over expression of P-selectin facilitates further attachment of EV allowing for further expression of its tissue factor.

The family of selectins and their ligands play roles in the metastatic process and possibly organ selectivity for metastasis. P-selectin facilitates the initial attachment, with subsequent other cellular adhesion molecules (CAMs) furthering the process, including interaction with platelet- and fibrinogen-causing clots with further anchoring of circulating malignant cells [127–131]. Increased adhesion for monocytes and leukocytes further accelerate the clotting process, leading to increased endothelial cell apoptosis and shear-induced endothelial cell loss causing exposure of subendothelial substances [132].

The angiocrinic function of endothelial cells has been reviewed describing its influence upon tumor growth, as well as organ regeneration and differentiation [121]. The endothelial cells preserve specific niches useful for selective stem cell implantation. This process is associated with inhibitory or stimulatory activities that effect downstream trophagens and cytokines that in turn regulate adoptive healing and metastatic processes [52, 54, 95, 119, 120, 133].

Annexin I and annexin V are cell-specific receptors for the exposed phosphatidyl serine on the EV surfaces, working synergistically with its PSGL-1 ligand for P-selectin [24, 41, 55, 134–136].

## Vascular ligand–receptor mapping

Combinatorial screenings with phage display can be used to identify peptides and proteins with high affinity and specificity for EV [137, 138]. The technique has been used to examine ligand–receptor interactions on the endothelial cells of blood vessels allowing selection of peptides that bind specifically to different vascular beds [123, 138–141]. This technique has been used for unbiased mapping of vascular diversity, the vascular ZIP codes [142, 143]. These ZIP codes are conceived to be the basis for specific ligand delivery on intravascular endothelium. Furthermore, it is conceived that these ZIP codes may vary in certain diseases, including cancers [142, 143]. They are evolving into site-specific targets for drug delivery for prevention of metastatic cell deposits and for the treatment of cancers [141, 142].

It appears possible that phosphatidyl serine receptor-mediated actions as assayed using the ZIP code identification may allow detection of differences in the receptor density based upon source of the endothelial cells and thus differences of EV adherence to those cells. This may add to our understanding of why certain veins are more likely to anchor circulating malignant cells and/or serve as a more effective platform have thrombosis.

## Blocking EV function

Creating agents that specifically interfere with the activity of selectin or their ligands is a major area of pharmacologic study [144]. Among already available agents are the HMG-CoA reductase inhibitors that reduce expression of P-selectin so as to reduce anchoring of EVs to the venous endothelium [76–81, 145]. Therapy with statin agents interferes with platelet microparticle (EV) attachment, seemingly causing a fall in the tissue factor expression and subsequent thrombin generation [79, 81, 145]. This may become a model for therapies in other diseases wherein EV play facilitating roles. Another example is a novel recombinant homodimer of annexin V, Diannexin, which binds to and shields phosphatidylserine thereby suppressing phosphatidylserine-mediated leukocyte and platelet attachments [146]. It has been used with success in animal models for preventing reperfusion injury after transplants of lung, muscle, kidney, liver, and islet cell transplants, and following myocardial infarct [147–151]. Quercetin-3-rutinoside blocks EV-related tissue factor activation and platelet aggregation. This latter agent may become a new class of anticoagulant [56, 105]. These same agents may reduce the potential of other EV functions, thus suppressing metastatic cell anchorage and tumor-related angiogenesis. Agents that block Tim4 activity, such as anti-Tim4 antibody, may have future roles, as may anti-P-selectin and P-selectin glycoprotein ligand -1 antibodies [35].

## Conclusions

While some details are known about the roles of circulating EV’s in the complex physiologic and pathophysiologic activities of endothelium there is much yet to be examined. It may be instructive to determine if EV from different source cells adhere to different organ-sourced endothelial cells with different densities and with differing endothelial cell response. It is unknown whether there is a steady state of microparticle attachment to endothelial surfaces in normal circumstances. Are there differences of any such density of adherence depending upon the site of origin of these veins? Is there an accelerated adherence of these particles in disease states that effect endothelial function and integrity? Is there enough of a difference in this density to explain difference in the rates of deep vein thrombosis in certain vessels and the placement of malignant masses in certain vessels but not others? Whether there are specific receptors that facilitate the incorporation of the EV’s membranous and submembranous contents into the interior of the adherent cells is another area in need of further study. Can cancer-derived EV facilitate the initiation of tumor metastasis cascade more efficiently than do intact circulating tumor cells?
